# Role of Dystrophic Calcification in Reparative Dentinogenesis After Rat Molar Pulpotomy

**DOI:** 10.3390/ijms26157130

**Published:** 2025-07-24

**Authors:** Naoki Edanami, Kunihiko Yoshiba, Razi Saifullah Ibn Belal, Nagako Yoshiba, Shoji Takenaka, Naoto Ohkura, Shintaro Takahara, Takako Ida, Rosa Baldeon, Susan Kasimoto, Pemika Thongtade, Yuichiro Noiri

**Affiliations:** 1 Division of Cariology, Operative Dentistry and Endodontics, Department of Oral Health Science, Niigata University Graduate School of Medical and Dental Sciences, Niigata 951-8126, Japan; dr.razi3187@gmail.com (R.S.I.B.); stakenaka@dent.niigata-u.ac.jp (S.T.); ohkura@dent.niigata-u.ac.jp (N.O.); takahara@dent.niigata-u.ac.jp (S.T.); tida@dent.niigata-u.ac.jp (T.I.); rosabaldeong@dent.niigata-u.ac.jp (R.B.); susangk@dent.niigata-u.ac.jp (S.K.); pemika.thongtade@dent.niigata-u.ac.jp (P.T.); noiri@dent.niigata-u.ac.jp (Y.N.); 2 Division of Oral Science for Health Promotion, Department of Oral Health and Welfare, Niigata University Graduate School of Medical and Dental Sciences, Niigata 951-8126, Japan; yoshiba.dent@niigata-u.ac.jp (K.Y.); nagako@dent.niigata-u.ac.jp (N.Y.)

**Keywords:** dystrophic calcification, osteopontin, dentin matrix protein-1, pulpotomy, reparative dentinogenesis

## Abstract

Vital pulp therapy with calcium hydroxide or mineral trioxide aggregate (MTA) rapidly induces dystrophic calcification and promotes the accumulation of two members of small integrin-binding ligand N-linked glycoproteins: osteopontin (OPN) and dentin matrix protein-1 (DMP1). However, the precise relationship between these initial events and their roles in reparative dentinogenesis remain unclear. This study aimed to clarify the relationship between dystrophic calcification, OPN and DMP1 accumulation, and reparative dentin formation. Pulpotomy was performed on rat molars using MTA or zirconium oxide (ZrO_2_). ZrO_2_ was used as a control to assess pulp healing in the absence of dystrophic calcification. Pulpal responses were evaluated from 3 h to 7 days postoperatively via elemental mapping, micro-Raman spectroscopy, and histological staining. In the MTA-treated group, a calcium-rich dystrophic calcification zone containing calcite and hydroxyapatite was observed at 3 h after treatment; OPN and DMP1 accumulated under the dystrophic calcification zone by day 3; reparative dentin formed below the region of OPN and DMP1 accumulation by day 7. In contrast, these reactions did not occur in the ZrO_2_-treated group. These results suggest that dystrophic calcification serves as a key trigger for OPN and DMP1 accumulation and plays a pivotal role in reparative dentinogenesis.

## 1. Introduction

The dentin–pulp complex has an inherent capacity for repair and regeneration. When the pulp is exposed due to dental trauma or caries, reparative dentin (RD) can form through the activity of newly differentiated odontoblast-like cells, provided that infection is controlled [1]. This capacity underlies the success of vital pulp therapies (VPTs).

VPT is increasingly recognized as a favorable alternative to conventional root canal treatment for the management of teeth with pulpal disease [2]. Although spontaneous pain and prolonged thermal sensitivity have traditionally been considered as hallmarks of irreversible pulpitis requiring root canal treatment [3], emerging evidence suggests that partial or full pulpotomy may be effective even in teeth presenting with these symptoms [4]. Compared with root canal treatment, VPT offers a less-invasive approach that reduces procedural complexity, preserves pulp vitality and function, and helps maintain the structural integrity of the tooth [5]. These benefits may lead to better long-term outcomes and enhanced preservation of natural teeth.

To further improve the clinical efficacy and predictability of VPT, it is essential to elucidate the mechanisms by which pulp capping agents promote tissue repair. Calcium hydroxide (CH) has traditionally been regarded as the gold standard for pulp capping owing to its strong antibacterial effects and capacity to stimulate dentin bridge formation [6]. However, CH has several drawbacks, including high solubility, poor long-term sealing ability, and the propensity to stimulate the formation of RD with tunnel defects, which may compromise the quality of the pulp seal and increase the risk of reinfection [7]. In recent years, mineral trioxide aggregate (MTA) has been widely used as a pulp capping material owing to its ability to effectively overcome the limitations of CH. MTA primarily comprises tricalcium and dicalcium silicates, with bismuth oxide for radiopacity, and small amounts of gypsum and other mineral phases [8]. When combined with water, it sets by forming calcium silicate hydrate, which ensures mechanical stability, and CH is produced as a byproduct, contributing to its bioactivity [8]. Compared with CH, MTA exhibits lower solubility, enhanced sealing ability, and greater mechanical strength [9]. Moreover, it induces the formation of a more homogeneous and structurally sound hard tissue barrier with fewer tunnel defects than CH [10]. Owing to its favorable physicochemical and biological properties, MTA is a suitable reference material for investigating the cellular and molecular mechanisms underlying pulp wound healing following VPT.

Previous studies have identified key early events in pulp wound healing. After CH or MTA is applied to the exposed pulp, noncollagenous proteins, such as osteopontin (OPN) and dentin matrix protein-1 (DMP1), accumulate at the exposed site prior to the appearance of odontoblast-like cells [11,12]. OPN and DMP1 are small integrin-binding ligand N-linked glycoproteins (SIBLING) and share common characteristics [13]. These proteins exhibit a high affinity for hydroxyapatite and regulate mineralization [14,15]. Furthermore, their arginine–glycine–aspartic acid motif binds to cell surface integrins, promoting cell adhesion and signaling [16,17]. Notably, strong expression of these proteins has been observed in the outermost layer of RD [11,12], indicating their involvement in odontoblast-like cell differentiation and early mineralization of RD.

Dystrophic calcification also occurs on the pulp wound surface during the initial healing phase after pulp capping with CH or MTA [18,19]. This process involves the deposition of calcium crystals on cellular debris and swollen collagen fibrils [18,20,21]. Although the exact role of dystrophic calcification in pulp tissue repair remains unclear, several studies suggest that it may contribute to the formation of RD. For example, Yoshiba et al. [18] and Tziafas et al. [22] demonstrated that fibronectin, a noncollagenous protein that promotes odontoblast-like cell differentiation [23], is adsorbed onto dystrophic calcification deposits in the dental pulp. Furthermore, Higashi and Okamoto [20] showed that RD forms in direct contact with the dystrophic calcification zone, indicating that dystrophic calcification serves as a scaffold or signaling source for the initiation of hard tissue regeneration.

The accumulation of OPN and DMP1 is presumed to be associated with dystrophic calcification, as they appear in the same locations and at similar time points during early pulp wound healing [11,12,18,19]. However, the nature of this association has yet to be fully elucidated. Moreover, the influence of these early events on RD formation is poorly understood, thus warranting further investigation.

Therefore, this study aimed to clarify the relationship between dystrophic calcification, OPN and DMP1 accumulation, and RD formation. To this end, pulpotomy was performed on rat molars using MTA or a bioinert material, zirconium oxide (ZrO_2_). ZrO_2_ served as a negative control to assess the pulp healing process in the absence of dystrophic calcification. The pulpal responses were subsequently evaluated through elemental mapping, micro-Raman spectroscopy, and histological staining.

This study hypothesized that dystrophic calcification is associated with the accumulation of OPN and DMP1 and contributes to the formation of RD.

## 2. Results

### 2.1. Dystrophic Calcification Beneath MTA

**Figure 1:**
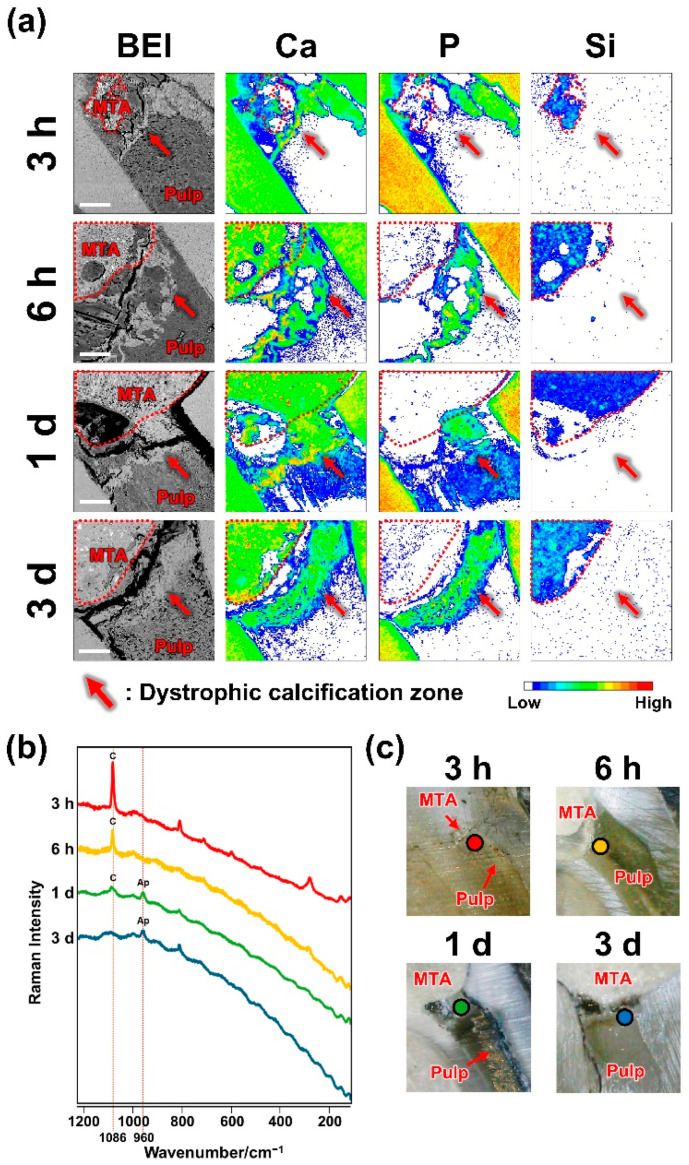
Dystrophic calcification after pulpotomy with mineral trioxide aggregate (MTA). (**a**) Backscattered electron images (BEIs) and elemental mapping images showing the distributions of calcium (Ca), phosphorus (P), and silicon (Si) at the pulp exposure site (453 × 453 μm). The MTA is outlined with red dotted lines. A Ca-rich dystrophic calcification zone was present during all observation periods. Scale: 100 μm. (**b**) Raman spectra of the pulp stump beneath the MTA. Raman peaks corresponding to hydroxyapatite (Ap) at 960 cm^−1^ and calcite (C) at 1086 cm^−1^ were detected. (**c**) Bright-field microscopic images of the specimens used for Raman spectroscopy. The colored dots in the bright-field images indicate the locations of the Raman measurements.

Using elemental mapping and Raman spectroscopy, we evaluated early mineral deposition beneath the MTA. A calcium-rich dystrophic calcification zone was observed beneath the silicon-rich MTA at 3 h, 6 h, 1 day, and 3 days (Figure 1, Table 1). The Raman spectra of the pulp under MTA exhibited peaks at 1086 and 960 cm^−1^, indicating the presence of calcite and hydroxyapatite [24], respectively (Figure 1).

### 2.2. Pulpal Responses After MTA Pulpotomy

Three complementary staining techniques were employed to evaluate pulpal healing responses. Hematoxylin and eosin (H&E) staining was used to qualitatively evaluate RD formation and to confirm the absence of excessive inflammatory response, particularly in the ZrO_2_ group. Immunohistochemistry enabled the time-resolved detection of OPN and DMP1 expressions. Double fluorescence staining with calcein blue and immunofluorescent antibodies was employed to determine the spatial association between mineralized deposits and protein localization.

**Figure 2:**
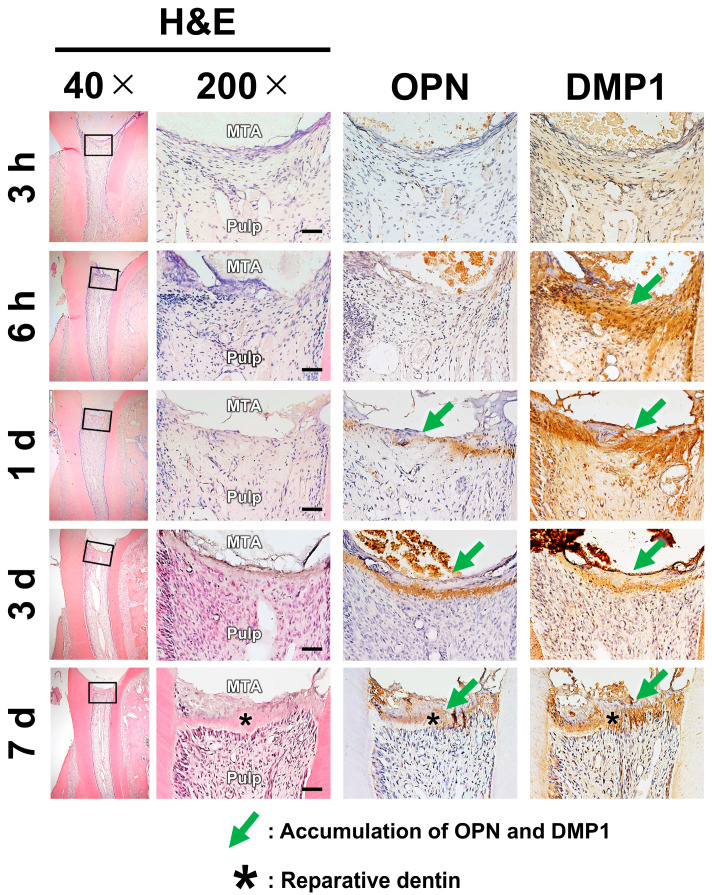
Osteopontin (OPN) and dentin matrix protein-1 (DMP1) expressions as well as reparative dentin formation following pulpotomy with mineral trioxide aggregate (MTA). Consecutive sections stained with hematoxylin and eosin (H&E) and antibodies against OPN and DMP1 are presented. DMP1 accumulation was detected at 6 h, whereas OPN accumulation was detected after 1 day. RD formation was detected beneath the OPN and DMP1 accumulation on day 7. Images were obtained at an original magnification of 40× (low magnification) and 200× (high magnification). The area indicated by a square in the low-magnification image is shown at a higher magnification. H&E: hematoxylin and eosin staining. Scale: 50 μm.

**Figure 3:**
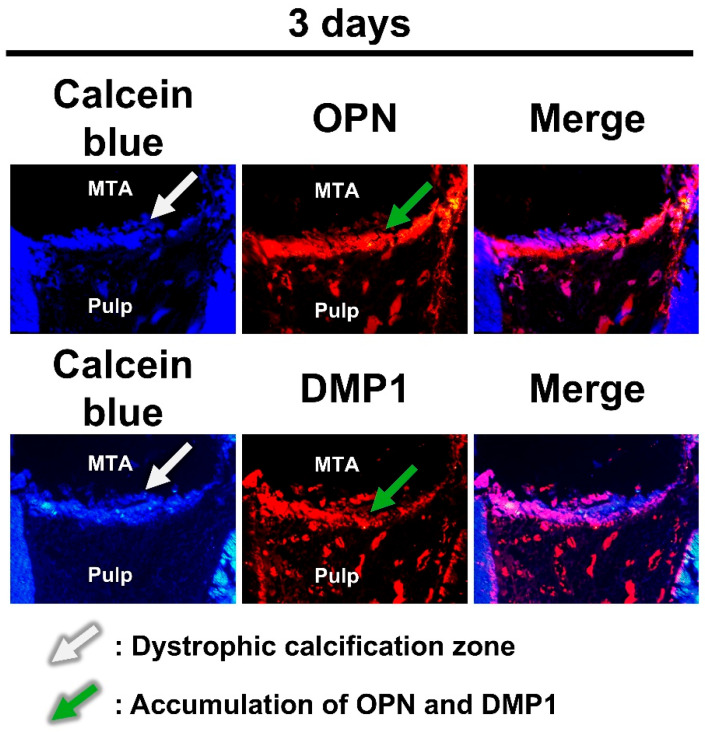
Spatial association between the dystrophic calcification zone and the accumulation of osteopontin (OPN) and dentin matrix protein-1 (DMP1). OPN and DMP1 immunoreactivities were directly localized beneath the calebin blue–positive dystrophic calcification zone. MTA: mineral trioxide aggregate.

H&E staining showed the presence of RD on day 7. The accumulation of OPN or DMP1 was not detected via immunostaining at 3 h. DMP1 was detected at 6 h, whereas OPN was detected after 1 day (Figure 2, Table 1). Double staining with calcein blue and immunofluorescent antibodies revealed OPN and DMP1 expressions directly beneath the calcein blue–positive dystrophic calcification zone (Figure 3).

### 2.3. Dystrophic Calcification and Pulpal Responses After ZrO_2_ Pulpotomy

To assess pulpal responses in the absence of dystrophic calcification, the same analytical methods, i.e., elemental mapping, histological staining, and immunohistochemistry, were used in the ZrO_2_ group.

**Figure 4:**
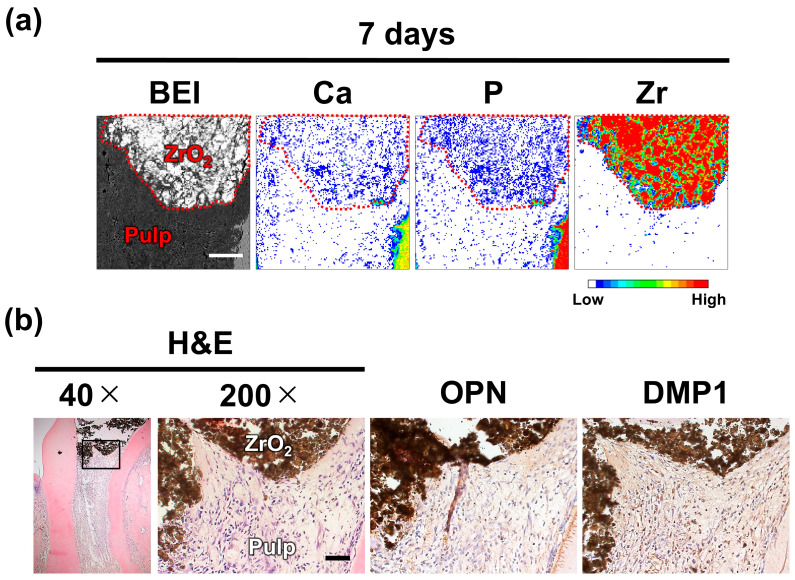
Dystrophic calcification and tissue responses in pulp treated with zirconium oxide (ZrO_2_). (**a**) A backscattered electron image (BEI) and elemental mapping images showing calcium (Ca), phosphorus (P), and zirconium (Zr) distributions in the pulp stump (453 × 453 μm). The ZrO_2_ is outlined with red dotted lines. No Ca-rich regions were detected beneath the ZrO_2_. Scale: 100 μm. (**b**) Hematoxylin and eosin staining (H&E) and immunohistochemical staining for osteopontin (OPN) and dentin matrix protein-1 (DMP1) were performed on consecutive sections. Only minimal inflammation was observed at the pulp amputation site, and neither OPN nor DMP1 accumulation was detected beneath ZrO_2_. Images were captured at original magnifications of 40× (low) and 200× (high). The area indicated by a square in the low-magnification image is shown at a higher magnification. Scale: 50 μm.

Elemental mapping revealed no calcium-rich zones under ZrO_2_. H&E staining revealed minimal to no inflammation in the pulp beneath ZrO_2_, and no RD formation was observed. No OPN or DMP1 accumulation was detected with immunostaining (Figure 4, Table 2).

## 3. Discussion

The results of this study elucidated the spatiotemporal distribution of dystrophic calcification, OPN and DMP1 accumulation, and RD formation after pulpotomy with MTA and ZrO_2_.

Dystrophic calcification is defined as the deposition of calcium crystals in degenerated or necrotic tissues, occurring despite normal serum calcium and phosphate levels [25]. The application of CH or MTA to exposed pulp rapidly induces calcium crystal precipitation in the pulp [18,19]. Based on ultrastructural analysis, calcium crystals formed under CH or MTA are associated with degenerated cells and swollen collagen, exhibiting characteristic features of dystrophic calcification [18,20,21]. CH and MTA are highly alkaline, causing tissue degeneration upon contact [1], and calcium release from CH and MTA promotes crystal nucleation and growth [26]. These factors likely contribute to dystrophic calcification in dental pulp.

Additionally, MTA forms a calcium silicate hydrate (CSH) gel during hydration. CSH gel is negatively charged, attracting calcium and phosphate ions and acting as nucleation sites for hydroxyapatite crystals [27,28]. Therefore, CSH gel formation by MTA may further promote dystrophic calcification by providing additional sites for mineral deposition in degenerated pulp tissue.

OPN and DMP1 are SIBLING family acidic glycoproteins. DMP1 is localized in the peritubular dentin and dental pulp, whereas OPN is found in predentin, mantle dentin, dentin–cementum interface, and tertiary dentin [29]. Previous studies have shown that their expression is upregulated in response to injury in the dentin–pulp complex, and that they colocalize in the superficial layer of RD following pulp capping with CH [12] or GaAlAs laser irradiation [30], indicating their involvement in reparative dentinogenesis.

Previous studies have demonstrated that dystrophic calcification occurs within 1 day in human [18] and canine teeth [20] after pulp capping with CH. In this study, elemental mapping detected a calcium-rich dystrophic calcification zone beneath MTA as early as 3 h after operation (Figure 1, Table 2). Moreover, micro-Raman spectroscopy confirmed the presence of calcite and hydroxyapatite in the zone (Figure 1), consistent with previous findings regarding calcite-like birefringent structures [31] and calcium–phosphorus crystals [19,20] in the pulp beneath CH or MTA.

The accumulation of OPN and DMP1 occurred later than dystrophic calcification (Table 2). Consistent with previous studies [11,12], DMP1 began accumulating after 6 h, whereas OPN accumulation became clear after 1 day. Furthermore, histological analysis of undecalcified sections revealed that areas of OPN and DMP1 accumulation were located just beneath the dystrophic calcification zone (Figure 3). Based on this spatiotemporal relationship, it is possible that dystrophic calcification induces the accumulation of OPN and DMP1.

The mechanism by which OPN and DMP1 accumulate adjacent to the dystrophic calcification zone remains unclear. One possible explanation is that OPN and DMP1 in tissue fluids are adsorbed onto hydroxyapatite within the dystrophic calcification zone. This hypothesis is supported by the high affinity of OPN [14] and DMP1 [15] for hydroxyapatite. Alternatively, OPN and DMP1 accumulation in the area adjacent to the dystrophic calcification zone may result from nanohydroxyapatite-induced upregulation of OPN and DMP1 expression in nearby cells. Supporting this possibility, previous studies have demonstrated that crystals in the dystrophic calcification zone are nanosized [18,19] and that nanohydroxyapatite enhances OPN and DMP1 expression in dental pulp stem cells [32,33]. These potential mechanisms warrant further investigation.

For this study, ZrO_2_ was selected owing to its well-established biological inertness. Previous studies have shown that ZrO_2_ exhibits negligible cytotoxicity and inflammatory responses when implanted in soft and hard tissues [34,35] and poses minimal risk of inducing hypersensitivity reactions or foreign body responses [36]. Moreover, contrary to MTA, ZrO_2_ does not release bioactive ions, such as calcium (Ca^2+^) or hydroxide (OH^−^), and does not induce hydroxyapatite formation under physiological conditions [37]. The inert nature of ZrO_2_ limits its interaction with surrounding tissues, making it suitable as a negative control in studies assessing material-induced tissue responses.

Although the MTA group underwent time-course analysis from 3 h to 7 days to track the sequential progression of dystrophic calcification, OPN and DMP1 accumulation, and RD formation, the ZrO_2_ group was examined only at the final time point (day 7). This is because the purpose of the ZrO_2_ group was not to monitor temporal changes but to determine whether these events occur in the absence of any bioactive effects from the capping material.

As expected, even after 7 days, the pulp tissue beneath ZrO_2_ exhibited no signs of dystrophic calcification. Furthermore, no accumulation of OPN or DMP1 was detected during the same period (Figure 4, Table 2). These results indicate that OPN and DMP1 accumulation in the MTA-treated specimens is not a spontaneous response to pulp exposure. Instead, it appears to be specifically induced by MTA, likely due to its ability to promote dystrophic calcification.

Compared with the MTA group, the ZrO_2_ group exhibited delayed RD formation. Specifically, all samples in the MTA group exhibited RD by day 7 (Table 1), whereas none of the day 7 samples in the ZrO_2_ group showed this change (Table 2). Importantly, this difference was not attributable to differences in inflammatory status, as both groups exhibited minimal or no inflammation at day 7. These findings are consistent with those of previous studies showing no RD in pulp capped with bioinert materials such as Teflon [38] or 4-META/MMA-TBB resin [39].

The differences in tissue responses following pulpotomy with MTA and ZrO_2_ may, at least in part, be attributed to the distinct capacities of these materials to induce dystrophic calcification. Dystrophic calcification deposits adsorb fibronectin [18,22], which promotes odontoblast-like cell differentiation [23]. Additionally, nanohydroxyapatite, which is likely present in the dystrophic calcification zone, induces odontoblastic differentiation of dental pulp stem cells [32]. Moreover, RD is continuously formed beneath the dystrophic calcification zone [20]. Therefore, the dystrophic calcification zone may serve as a substrate for odontoblast-like cell differentiation and promote the formation of RD.

OPN and DMP1 accumulated at the pulp exposure site, and this was followed by the formation of RD in the MTA group (Table 1). However, these changes were not observed in the ZrO_2_ group (Table 2), suggesting that the accumulation of OPN and DMP1 may be a prerequisite for the formation of RD. Recombinant DMP1–impregnated collagen matrix has been shown to induce odontoblast-like cell differentiation in rat molars [40]. Similarly, OPN has been reported to stimulate the odontoblastic differentiation of human dental pulp cells in vitro [41]. Therefore, the presence of OPN and DMP1 at the pulp exposure site may facilitate the differentiation of odontoblast-like cells and promote the formation of RD. However, further research is needed to elucidate their specific roles at pulp exposure sites.

This study has several limitations. First, the observation period was limited to postoperative day 7. Although this time frame was selected to capture the early events associated with dystrophic calcification and the initial OPN and DMP1 expressions, it precludes the evaluation of long-term tissue responses such as RD maturation. Second, although this study mainly focused on OPN and DMP1 accumulation, other extracellular matrix (ECM) remodeling events, such as fibrillin-1 degradation [42] and increased collagen production [43,44], have also been reported to precede reparative dentinogenesis. However, whether these ECM changes are regulated by dystrophic calcification remains unclear. Third, this study exclusively used ProRoot MTA as the pulp capping material; however, many other tricalcium silicate–based materials are currently available for clinical use, including bismuth-free formulations to reduce cytotoxicity and discoloration [45] as well as materials with accelerated setting reactions or enhanced handling characteristics [46]. However, it is undetermined whether these alternative materials induce dystrophic calcification and comparable pulpal responses similar to MTA. Fourth, the sample size for each analysis was relatively small (n = 2–6), which may limit the generalizability of the findings. Collectively, more studies with longer follow-up, broader ECM analysis, larger sample sizes, and different materials are warranted to better understand the mechanisms of pulp healing and confirm the present findings.

In conclusion, dystrophic calcification occurred at an early stage after MTA pulpotomy, followed by OPN and DMP1 accumulation and the formation of RD. These processes did not occur in ZrO_2_-treated specimens. Our results suggest that dystrophic calcification triggers OPN and DMP1 accumulation and RD formation.

## 4. Materials and Methods

### 4.1. Materials

Two materials were utilized for pulpotomy: ProRoot MTA (Dentsply Sirona, York, PA, USA; Lot No. 333803) and ZrO_2_ (Wako, Osaka, Japan; Lot No. PAG2868). ProRoot MTA was prepared according to the manufacturer’s protocols. The powder was mixed with sterile distilled water at a powder-to-liquid ratio of 3:1 (*w*/*w*) on a silicone mixing pad using a plastic spatula until a putty-like consistency was achieved. Similarly, ZrO_2_ powder was mixed with sterile distilled water at a powder-to-liquid ratio of 5:1 (*w*/*w*) to produce a cohesive putty.

### 4.2. Pulpotomy Procedures

All animal experiments were approved by the Niigata University Animal Experimentation Committee (approval no. SA00903) and conducted according to relevant guidelines and regulations. The experiments included 58 male Wistar rats (Clea Japan, Tokyo, Japan) aged 8 weeks old. After the induction of anesthesia via intraperitoneal injection of medetomidine hydrochloride, midazolam, and butorphanol (Wako), pulpotomy was performed on the left maxillary first molar under a 20× magnification using a surgical microscope (S9D; Leica, Wetzlar, Germany). Specifically, the pulp chamber was accessed using a tungsten carbide bur (E0123 size 008; Dentsply), and the coronal pulp tissue was removed with the same bur. The exposed pulp stump was irrigated with 2.5% sodium hypochlorite (Neo Dental Chemical Products, Tokyo, Japan) and 3% hydrogen peroxide (Yoshida Pharmaceutical, Tokyo, Japan), followed by rinsing with saline (1 mL each), in accordance with previous studies [11,12]. Following hemostasis, MTA or ZrO_2_ was placed on the pulp stump, and the cavity was sealed with a flowable composite resin (Beautifil Flow; Shofu, Kyoto, Japan) using a bonding system (Clearfil Universal Bond Quick; Kuraray, Tokyo, Japan). The opposing lower first molars were extracted at the time of surgery to prevent fracture of the upper first molars. After surgery, the animals’ general health status was monitored daily by veterinary technicians. No signs of distress or abnormal behavior was observed throughout the observation period. At designated time points, 3 h, 6 h, 1 day, 3 days, and 7 days after pulpotomy, the animals were euthanized via carbon dioxide inhalation, and the maxillae containing the treated upper first molars were harvested for analysis.

### 4.3. Elemental Mapping and Micro-Raman Spectrometry

Specimens from the MTA (3 h, 6 h, 1 day, and 3 days; n = 4 per time) and ZrO_2_ (7 days; n = 4) groups were analyzed. The sample size was based on previous studies using similar models for qualitative analyses [47,48]. Specimens were fixed in 2.5% glutaraldehyde (Wako) buffered with 60 mmol/L HEPES (Dojindo Laboratories, Kumamoto, Japan), dehydrated in ethanol and acetone, embedded in methyl methacrylate (MMA) resin (Osteoresin; Wako), and ground using a rotary grinder (Vector Power Head; BUEHLER, Lake Bluff, IL, USA). The exposed surfaces containing the capping material and the pulp stump were gold-coated and analyzed for elemental distribution using an electron probe microanalyzer (EPMA1601; Shimadzu, Kyoto, Japan). The EPMA accelerating voltage was 15 kV, step size was 3 μm, and sampling time was 0.1 s per point. After EPMA analysis, the specimens were subjected to micro-Raman spectroscopy. The gold coating was removed by polishing with diamond pads (Struers, Champigny-sur-Marne, France). Subsequently, Raman spectra were obtained from the pulp stump beneath the MTA, targeting areas with dystrophic calcification. Measurements were performed using a micro-Raman spectrometer (NRS-3100; JASCO, Tokyo, Japan) equipped with a 100× objective lens. A 532 nm laser served as the excitation source, with an output power of 7.4 mW. To minimize thermal noise and enhance signal quality, the CCD detector was cooled to −50.0 °C.

### 4.4. Histological Staining of the Decalcified Sections

Specimens from the MTA (3 and 6 h and 1, 3, and 7 days; n = 6 per time point) and ZrO_2_ (7 days; n = 6) groups were analyzed. The sample size was based on previous studies using similar models for qualitative analyses [49,50]. After fixation with 4% paraformaldehyde (Wako), the specimens were demineralized in EDTA solution (OsteoSoft; Merck, Darmstadt, Germany), dehydrated in ethanol, cleared with xylene, embedded in paraffin, and sectioned (5 µm) using a microtome (HistoCore Multicut R; Leica, Wetzlar, Germany). Subsequently, the sections were stained with H&E (Wako) to evaluate the timing of RD formation and pulpal inflammation, particularly in the ZrO_2_ group, confirming the material’s biological inertness. Immunohistochemistry was performed using the primary antibodies listed in Table 3, following a previous protocol [43]. The staining results were qualitatively assessed by a trained observer in a blinded manner, with no knowledge of the group assignment or time point during the analysis.

### 4.5. Histological Staining of the Nondecalcified Sections

MTA specimens (3 days; n = 2) were fixed in phosphate-free 10% neutral formalin (Mutoh Chemical, Tokyo, Japan), dehydrated in ethanol and acetone, and then embedded in MMA resin (Technovit 9100; Kulzer, Wehrheim, Germany) via acetone evaporation [51]. Next, the embedded samples were cut into 3 µm sections using a hard tissue microtome (HistoCore Multicut R) and double-stained with calcein blue, a fluorescent dye that labels calcium, and an immunofluorescent antibody for OPN or DMP1. Sections were deresinized in methyl ethyl ketone and acetone, stained with calcein blue (10 mg/mL, 10 min; Wako), temporarily mounted, and imaged using a fluorescence microscope (Eclipse E800; Nikon, Tokyo, Japan). After imaging, the sections were subjected to decalcification with EDTA (OsteoSoft) for 20 min, antigen retrieval with hyaluronidase (Wako) at 37 °C for 20 min, and blocking with goat serum (Jackson Immuno Research Laboratories, West Grove, PA, USA) for 5 min, followed by overnight incubation with a primary antibody for OPN or DMP1 and then 1 h incubation with a secondary antibody (Alexa Fluor 546-conjugated anti-rabbit IgG; Invitrogen, Carlsbad, CA, USA). Subsequently, the sections were mounted again and reimaged. The resulting images were aligned and merged using Fiji (ImageJ 1.53t; National Institutes of Health, Bethesda, MD, USA) [52].

## Figures and Tables

**Table 1:** Pulpal responses after pulpotomy with mineral trioxide aggregate (Positive/Total).

	3 h	6 h	1 Day	3 Day	7 Day
DC	4/4	4/4	4/4	4/4	-
OPN	0/6	0/6	2/6	6/6	6/6
DMP1	0/6	2/6	6/6	6/6	6/6
RD	0/6	0/6	0/6	0/6	6/6

The presence or absence of dystrophic calcification (DC) was determined based on elemental mapping results. Osteopontin (OPN) and dentin matrix protein-1 (DMP1) accumulation were evaluated using immunohistochemical staining, whereas reparative dentin (RD) was assessed through hematoxylin and eosin staining.

**Table 2:** Pulpal responses after pulpotomy with zirconium oxide (positive/total).

	7 Day
DC	0/4
OPN	0/6
DMP1	0/6
RD	0/6

The presence or absence of dystrophic calcification (DC) was determined based on elemental mapping results. Osteopontin (OPN) and dentin matrix protein-1 (DMP1) accumulation were evaluated using immunohistochemical staining, whereas reparative dentin (RD) was assessed through hematoxylin and eosin staining.

**Table 3:** Primary antibodies used in this study.

Antibody (Catalog Number)	Concentration	Manufacturer
Rabbit polyclonal anti-osteopontin antibody (18628)	1:300	Immuno-Biological Laboratories, Gunma, Japan
Rabbit polyclonal anti-dentin matrix protein-1 antibody (M176)	1:300	Takara Bio, Shiga, Japan

## Data Availability

Relevant data from this study are available upon reasonable request to the corresponding author.

## Abbreviations

The following abbreviations are used in this manuscript:

BEI	Backscattered electron image
CH	Calcium hydroxide
CSH	Calcium silicate hydrate
DMP1	Dentin matrix protein-1
ECM	Extracellular matrix
EPMA	Electron probe microanalyzer
H&E	Hematoxylin and eosin
MMA	Methyl methacrylate
MTA	Mineral trioxide aggregate
OPN	Osteopontin
RD	Reparative dentin
SIBLING	Small integrin-binding ligand N-linked glycoproteins
ZrO2	Zirconium Oxide
